# Seed banks alter the molecular evolutionary dynamics of *Bacillus subtilis*

**DOI:** 10.1093/genetics/iyac071

**Published:** 2022-05-02

**Authors:** William R Shoemaker, Evgeniya Polezhaeva, Kenzie B Givens, Jay T Lennon

**Affiliations:** Department of Biology, Indiana University, Bloomington, IN 47405, USA; Department of Ecology and Evolutionary Biology, UCLA, Los Angeles, CA 90095, USA; Department of Biology, Indiana University, Bloomington, IN 47405, USA; Department of Ecology and Evolutionary Biology, UCLA, Los Angeles, CA 90095, USA; Luddy School of Informatics, Computing, and Engineering, Indiana University, Bloomington, IN 47408, USA; Department of Biology, Indiana University, Bloomington, IN 47405, USA

**Keywords:** experimental evolution, dormancy, *Bacillus*, seed banks, convergent evolution, microbial evolution

## Abstract

Fluctuations in the availability of resources constrain the growth and reproduction of individuals, which subsequently affects the evolution of their respective populations. Many organisms contend with such fluctuations by entering a reversible state of reduced metabolic activity, a phenomenon known as dormancy. This pool of dormant individuals (i.e. a seed bank) does not reproduce and is expected to act as an evolutionary buffer, though it is difficult to observe this effect directly over an extended evolutionary timescale. Through genetic manipulation, we analyze the molecular evolutionary dynamics of *Bacillus subtilis* populations in the presence and absence of a seed bank over 700 days. The ability of these bacteria to enter a dormant state increased the accumulation of genetic diversity over time and altered the trajectory of mutations, findings that were recapitulated using simulations based on a mathematical model of evolutionary dynamics. While the ability to form a seed bank did not alter the degree of negative selection, we found that it consistently altered the direction of molecular evolution across genes. Together, these results show that the ability to form a seed bank can affect the direction and rate of molecular evolution over an extended evolutionary timescale.

## Introduction

Nature is rarely static. Temporal fluctuations in abiotic and biotic environmental factors often reduce the rate that an organism can grow and reproduce. To contend with such fluctuations, many species enter a reversible state of reduced metabolic activity, an adaptation known as dormancy (Lennon and Jones 2011). In this state, individuals can endure environmental stressors until they subside, a temporary cessation of short-term reproductive efforts to increase long-term reproductive gains. This evolutionary trade-off, and the life-history strategies through which it is implemented, has received substantial attention by means of theoretical (Venable and Lawlor 1980; Venable and Brown 1988; Jones and Lennon 2010; Vitalis *et al.* 2013; Buoro and Carlson 2014; Rubio de Casas *et al.* 2015) and empirical investigations (Hairston *et al.* 1999; Huang *et al.* 2010; Tellier *et al.* 2011), spurred in part by the observation that dormancy has independently evolved multiple times across the tree of life (Guppy and Withers 1999; Willis *et al.* 2014; Shoemaker and Lennon 2018).

While the trade-off aspect of dormancy has been of considerable historical interest, life-history traits do not operate in a population genetic vacuum. The fitness benefit of a life-history trait is often a consequence of its effect on a given birth–death process (Doebeli *et al.* 2017), population dynamics that sequentially alter the dynamics and fates of genetic variants. The ability to enter a dormant state is no exception. The accumulation of dormant individuals within a system can result in the formation of seed banks (Shoemaker and Lennon 2018), demographic structures that can reshape the molecular evolutionary dynamics of a population.

Seed banks primarily alter the molecular evolutionary dynamics of a population through 2 means. First, the ability to enter a dormant state dampens the accumulation of de novo genetic diversity as well as the fluctuations of variants over their sojourn times, as dormant individuals do not reproduce and the vast majority of mutations are typically acquired during the process of genome replication (Kunkel 2004; Lennon *et al.* 2021). Second, seed banks can act as reservoirs of genetic and phenotypic diversity. These reservoirs reduce the efficiency of natural selection (Schoen *et al.* 1998; Blath *et al.* 2015; Koopmann *et al.* 2017), dampen the loss of genetic diversity due to random genetic drift (Kaj *et al.* 2001; Blath *et al.* 2015, 2016; Heinrich *et al.* 2018; Tellier 2019; van Gestel *et al.* 2019), and permit the retention of deleterious variants (Ellner and Hairston 1994; Nunney 2002). Together, the presence of a seed bank reduces the rate of molecular evolution while increasing the maximum amount of genetic diversity that can be retained. Furthermore, because the ability to form a seed bank is the result of a life-history strategy maintained by natural selection, it is possible that the formation of seed banks restricts the targets of molecular evolution, constraining the direction of evolution as well as its rate. While substantial progress has been made toward developing mathematical models that describe these patterns within the discipline of theoretical population genetics (Kaj *et al.* 2001; Blath *et al.* 2015, 2016; Koopmann *et al.* 2017; van Gestel *et al.* 2019), there remains a comparative lack of experimental tests of central predictions.

The role of seed banks as an evolutionary buffer means that time is an essential factor when considering an appropriate empirical system. Namely, if the per-generation rate of change of genetic diversity is reduced by a given amount, it is often necessary to observe an additional proportionate number of generations. This constraint makes it challenging to directly observe seed bank dynamics over extended evolutionary timescales. Given their short generation times, large population sizes, propensity for rapid adaptation, and the prevalence of dormancy among their lineages (Lennon and Jones 2011), microorganisms are an ideal group of organisms to characterize the extent that dormancy alters molecular evolutionary dynamics. In addition, certain lineages of microorganisms have evolved the ability to form complex protective structures (i.e. endospores) that allow them to survive and form long-lasting seed banks (Weller and Wu 2015; Shoemaker, Jones *et al.* 2021). While these structures are not the only means by which microorganisms can enter a dormant state (Sachidanandham and Yew-Hoong Gin 2009), their existence provides a means through which the formation of seed banks can be genetically manipulated (Siebring *et al.* 2014). Furthermore, while evolution experiments have been performed using seed bank forming microorganisms (Zeigler and Nicholson 2017), questions pertaining to dormancy have primarily been restricted to examining the phenotypic decay of endospore formation via the acquisition of de novo mutations under relaxed selection (Maughan *et al.* 2006, 2007, 2009), whereas the effect of endospore formation on the molecular evolutionary dynamics of microorganisms has remained relatively unexplored. In this study, we examined the molecular evolutionary dynamics of *Bacillus subtilis* populations that differ in their ability to form protective endospores, a nonreproductive structure that is the primary mechanism through which this species enters a dormant state to form a seed bank. To manipulate seed bank formation, we replenished the resources of dormancy-capable and incapable populations via serial dilution every 1, 10, and 100 days, a set of transfer regimes that allowed us to examine the effect of dormancy across a range of energy availabilities. Replicate populations were maintained for over 700 days, generating a molecular fossil record, which was reconstructed to determine how the presence of a seed bank altered the trajectories of de novo mutations. We then recapitulated the dynamics we observed using simulations based on a stochastic model of molecular evolution in dormancy-capable populations. Finally, we identified the sets of genes that were enriched for mutations within each transfer regime for dormancy-capable and incapable populations, allowing us to quantify parallel evolution among replicate populations as well as the degree of divergent evolution between dormancy-capable and incapable populations.

## Materials and methods

### Mutant construction

To manipulate endospore formation we deleted *spo0A*, the master regulatory gene for sporulation pathways in *B. subtilis*. Gene deletion was performed using Gibson assembly of PCR amplified dsDNA fragments upstream and downstream of *spo0A* (Supplementary Table 1). Purified ligated plasmid was transformed into *Escherichia*  *coli* DH5*α* and plasmid DNA was purified from cultured positive transformants. Purified plasmid product was transformed into *E.*  *coli* TG1, positive transformants were selected, and plasmid DNA was purified before a single *B. subtilis* NCIB 3610 colony was grown in medium containing a purified plasmid aliquot and identified using antibiotic plating. Transformation was confirmed via Sanger sequencing of PCR products and loss of antibiotic resistance was confirmed via antibiotic plating (see Supplementary Methods for additional detail).

### Evolution experiment

#### Fitness assay

We performed fitness assays to determine the degree that the fitness effect of endospore formation varied across transfer regimes. At mid-exponential phase, equal ratio aliquots of *B. subtilis* WT and *B. subtilis* Δ*spo0A* cultures were transferred to 3 replicate flasks with fresh medium. Aliquots were frequently sampled from each flask in a sterile biosafety cabinet over 100 days. Strains were distinguishable by the morphology of their colony forming units (CFUs) on agar plates, which were used to obtain estimates of the size of the population (*N*) in a flask via serial dilution. To make sure the CFU morphology of a given strain was stable, we maintained 5 replicate populations of WT and Δ*spo0A* strains in separate flasks for 100 days and plated each population every 10 days. We found zero evidence that WT CFUs converted to Δ*spo0A* morphology or that Δ*spo0A* CFUs converted to WT morphology, allowing us to use colony morphology to distinguish between strains for a 100-day fitness assay. The relative log fitness of Δ*spo0A* after *t* days was defined as

(1) $$X(t)\equiv \;\text{log}\;\left[\frac{N_{\Delta \mathrm{spo}0A}(t)}{N_{\text{WT}}(t)}\cdot \frac{N_{\text{WT}}(0)}{N_{\Delta \mathrm{spo}0A}(0)}\right].$$

Because we were measuring populations that exited their exponential phase of growth, we do not know how many generations accrued over the course of the fitness assay. Therefore, we chose to examine *X* at each given time point rather than attempt the typical fitness per-generation estimate $\Delta X=\frac{1}{\Delta \tau }\cdot X(t)$.

#### Transfer protocol, sequencing, and variant calling

To evaluate how environmental conditions interacted with the ability to form seed banks, we manipulated energy limitation by extending the time between transfers for microbial populations. We performed our energy-limited evolution experiments using *B.*  *subtilis* NCIB 3610 Δ*spo0A* (ASM205596v1) using a previously described methodology (Shoemaker, Jones *et al.* 2021). To briefly summarize, a single colony was isolated from each strain and grown overnight in 10 mL of PYE medium with 0.2% glucose and 0.1% casamino acids in a 50-mL Erlenmeyer flask and split into replicate populations. Five replicates were transferred as 1 mL aliquots into 9 mL of medium every 1, 10, or 100 days for 700 days at 25°C and 250 RPM. All replicate populations were cryopreserved every 100 days by mixing biomass with 20% glycerol solution. These samples were flash-frozen in liquid nitrogen and stored at −80°C. Biomass for DNA extraction was collected from all replicate populations every 100 days (Supplementary Fig. 1). Populations were regularly plated to test for contamination. An identical experiment was concurrently run with the WT strain, which was previously described (Shoemaker, Polezhaeva *et al.* 2021). To estimate the number of endospore CFUs, we performed heat treatment at 65°C on aliquots of cultures to kill vegetative cells. CFU counts of vegetative cells were then back-calculated using total CFU counts from nonheat-treated culture (i.e. ${\text{CFU}}_{\text{veg}}={\text{CFU}}_{\text{all}}-{\text{CFU}}_{\text{spore}}$).

In order to compare quantities that contain time as a unit between treatments, it is necessary to first identify an appropriate unit of time. For evolution, the generation is the fundamental unit of time. In a serially transferred batch culture experiment, the vast majority of cellular divisions, and, as a consequence, generations, accrue while the population is in its exponential phase of growth. Thus, starting with an initial size *N_i_*, a population will reach the final size *N_f_* over a window of time Δt with per-unit time growth rate *r* as $N_{f}=N_{i}\;\text{exp}[r\Delta t]$. By setting r=log(2), we have defined growth as a process of binary cellular division, a per-generation growth rate that, consequently, redefines our length of arbitrary time Δt as the number of generations that have surpassed (Δτ). After exchanging these variables, the growth of the population can be described as $N_{f}=N_{i}2^{\Delta \tau }$. Using this result, we can define the number of generations that surpassed within a given transfer cycle as $\Delta \tau ={\;\text{log}\;}_{2}(N_{f}/N_{i})$, a standard formula for calculating the number of generations in microbial evolution experiments (Powell 1958; Lenski 2017; Cooper 2018).

If a population remains in its exponential phase of growth at the end of a transfer cycle or if the decline in population size is negligible once exponential growth has halted, then the ratio of the final and the initial size of the population is approximately equal to the serial transfer dilution factor ($\frac{N_{f}}{N_{i}}\approx \text{dilutionfactor}$). Using this approximation, the 1:10 dilution ratio used in this experiment suggests that $\Delta \tau ={\;\text{log}\;}_{2}(\text{dilutionfactor})={\;\text{log}\;}_{2}(10)\approx 3.3$ accrued over a transfer cycle. However, a substantial number of cells died in the 10- and 100-day transfer regimes over a transfer cycle for both WT and Δ*spo0A* strains, meaning that the final size of a population (*N_f_*) relative to its initial size immediately after a transfer (*N_i_*) was less than a dilution factor of 10 ${\;\text{log}\;}_{2}(N_{f}/N_{i})>{\;\text{log}\;}_{2}(\text{dilutionfactor})$. Thus, estimates of Δτ obtained using the dilution factor will underestimate the true number of generations. To account for this discrepancy, we calculated the number of generations per-transfer using estimates of *N_f_* and *N_i_* (Table 2). Estimates of *N* were obtained via CFU counts. These estimates were used for calculating all quantities that contain “generations” as a unit (Equations 3 and 4b).

**Table 1.** We performed Wright–Fisher simulations to validate empirical distributions of measures calculated from mutation trajectories.

Event	Metabolic state	Pr[event]
(1) Change of metabolic state	Active	*c*/*A*
	Dormant	*c*/*D*
(2) Mutation	Active	$1/L_{\text{genome}}U_{b}$
	Dormant	ø
(3) Reproduction	Active	$e^{s_{i}(\tau )}/\sum_{j}^{A}e^{s_{j}(\tau )}$
	Dormant	ø

Each generation consisted of 3 steps: (1) change of metabolic state, where active individuals enter the seed bank with probability $\frac{c}{A}$ and dormant individuals are resuscitated with probability $\frac{c}{D}$, (2) mutation among active individuals with a per-individual probability of *U_b_*, and (3) the reproduction of active individuals. Reproduction was simulated as a multinomial sampling process, where the probability that a given individual reproduces is the exponential raised to the power of said individual’s selection coefficient at the current generation ($s_{i}(\tau )=X_{i}(\tau )-\bar{X}(\tau )$).

**Table 2.** To manipulate energy-limitation, we extended the time between resource replenishment via serial transfer, resulting in transfer regimes of 1, 10, and 100 days.

Transfer regime	Strain	$N_{f}/N_{i}\pm S.E.$	Gens. per-transfer, Δτ±S.E.	Total gens., *τ*
1 day	WT	10.0	3.33	3,300
	Δ*spo0A*	10.0	3.33	2,330
10 days	WT	210.0 ± 48.6	7.61 ± 0.313	761
	Δ*spo0A*	20.0 ± 1.80	4.31 ± 0.138	302
100 days	WT	425 ± 104	8.55 ± 0.458	85.5
	Δ*spo0A*	17,100±6,110	13.6±0.764	81.6

The decline in population size over a single transfer cycle was inconsequential for the 1-day transfer regime, allowing us to use the dilution factor to estimate the number of generations. However, this approximation could not be extended to the 10- and 100-day transfer regimes for both the WT and Δ*spo0A* strains, implying that the ratio of final and initial population sizes ($N_{f}/N_{i}$) was greater than the dilution factor by a varying degree across strain-transfer regime combinations. Under a standard serial dilution regime, the number of generations was estimated as the binary logarithm of $N_{f}/N_{i}$. By obtaining estimates of *N_i_* for the 10- and 100-day transfer regimes, we obtained appropriate estimate of the number of generations that accrued per-transfer (Δτ). Using these per-generation estimates, we calculated the total number of generations (*τ*), a quantity that represents the evolutionary timescale of a given strain/transfer regime combination.

While this estimate is more accurate than the one based on dilution factors, it does not account for the number of generations that accrued between transfers after a population exited its exponential phase of growth. This unknown number of generations likely occurs as a result of living cells using dead cells as a resource for reproduction. While there is no reliable approach to calculate the number of generations once a population has exited exponential growth, known as “cryptic growth” (Mason and Hamer 1987; Banks and Bryers 1990; Shoemaker, Polezhaeva *et al.* 2021), evidence suggests that this number is likely low (Bradley *et al.* 2018; Shoemaker, Jones *et al.* 2021) and can be safely neglected for the purpose of this study.

DNA extraction, library preparation, and pooled population sequencing were performed on all Δ*spo0a* and WT timepoints for all replicate populations as previously described (Shoemaker, Polezhaeva *et al.* 2021). The first 20 bp of all reads were trimmed and all read pairs where at least 1 pair had a mean Phred quality score ($=-10{\;\text{log}\;}_{10}P$; P= probability of an incorrect base call) less than 20 were removed via cutadept v1.9.1 (Martin 2011). Candidate variants were identified using a previously published approach (Good *et al.* 2017) that relied on alignments generated from breseq v0.32.0 (Deatherage and Barrick 2014), which was modified as previously described (Shoemaker, Polezhaeva *et al.* 2021).

#### Mutation trajectory analyses

We estimated the frequency of the *m*th mutation candidate in the *p*th population at the *t*th timepoint using the naive estimator ${\hat{f}}_{\mathrm{pmt}}\equiv F_{\mathrm{pmt}}/G_{\mathrm{pmt}}$, where *F_pmt_* and *G_pmt_* are the total number of reads containing the alternate allele and the total depth of coverage, respectively. We examined the accumulation of mutations by time *t* in the *p*th population as the sum of derived allele frequencies

(2) $$M(t)\equiv \sum_{m}{\hat{f}}_{\mathrm{pmt}}.$$

Given that the log of *M*(*t*) over time often appeared to saturate in this study as well as in previous studies (Good *et al.* 2017; Shoemaker, Polezhaeva *et al.* 2021), we modeled the relationship between *M*(*t*) and *t* using the following equation:

(3) $${\;\text{log}\;}_{10}M(t^{*})={\;\text{log}\;}_{10}M(t^{*}=0)+{\left[{\;\text{log}\;}_{10}M\right]}_{\text{max}}\frac{t^{*}}{t_{1/2}^{*}+t^{*}},$$

where ${\left[{\;\text{log}\;}_{10}M\right]}_{\text{max}}$ is the maximum value of ${\;\text{log}\;}_{10}M(t^{*})$ and $t_{1/2}^{*}$ is the value of $t^{*}$ where ${\;\text{log}\;}_{10}M(t^{*})$ is half of ${\left[{\;\text{log}\;}_{10}M\right]}_{\text{max}}$. The variable $t^{*}$ represents the shift in time so that Equation (3) reduces to the intercept parameter (${\;\text{log}\;}_{10}M(0)$) at the first temporal sample, in this case, $t^{*}=t-100\text{days}$. We then multiplied $t_{1/2}^{*}$ by the estimated minimum number of per-day generations, the product of which we define as $\tau_{1/2}$. While this model is phenomenological in that we do not posit a microscopic mechanism, much like the Michaelis–Menten kinetic model from which it is derived (Transtrum and Qiu 2016), it effectively captures the hyperbolic pattern. Numerical optimization was performed over 54 initial conditions using the Broyden–Fletcher–Goldfarb–Shanno (BFGS) algorithm in Python using statsmodels (Seabold and Perktold 2010). Estimates of the probability of extinction of alleles that were detected Pr[Extinct|Detected] for a given strain-transfer regime combination were calculated as the number of mutations where an extinct state was inferred by the end of the experiment divided by the total number of detected mutations.

We examined 3 different measures to determine how the ability to enter a dormant state affected molecular evolutionary dynamics, defined as

(4a) $$f_{\text{max}}\equiv \text{max}\left(\left\{f(t):t=1,\ldots ,T\right\}\right)$$

 

(4b) $$\frac{\left|\Delta f\right|}{\Delta \tau }\equiv \frac{\left|f(\tau +\Delta \tau )-f(\tau )\right|}{\Delta \tau }$$

 

(4c) $$\frac{f(\tau +\Delta \tau )}{f(\tau )}.$$

First, $f_{\text{max}}$ is the maximum estimated frequency of a given mutation over *T* observations. The majority of replicate populations had *T *=* *7 observations. Because certain 100-day transfer regime replicates populations had *T *=* *6 observations, we only used these 6 observations for estimates of $f_{\text{max}}$ for all replicate populations. Second, |Δf|/Δτ is the magnitude of change in *f* between 2 observations. Finally, f(τ+Δτ)/f(τ) is the direction of change for *f* between 2 observations. Given the absence of information about physical linkage between mutations in pooled data, we calculated a measure of statistical dependence based on a previously described implementation (McDonald *et al.* 2016). As a measure reflective of the rate of recombination, we calculated the Pearson correlation coefficient between all pairs of mutations that were simultaneously segregating (i.e. 0<f<1) for at least 3 timepoints for each replicate population.

We compared the empirical cumulative distribution functions of WT and Δ*spo0A* for all measures for each transfer regime using the Kolmogorov–Smirnov (KS) test. To identify fixed mutations, we used a previously published hidden Markov model (Good *et al.* 2017) to infer whether a given mutation eventually became fixed within a replicate population over the course of the experiment. The ratio of nonsynonymous to synonymous polymorphic mutations *pN*/*pS* was calculated in each population, where the total number of observed mutations of each class was weighted by the relative frequency of nonsynonymous and synonymous sites in all genes in the genome.

### Parallelism at the gene level

We identified potential targets of selection by examining the distribution of nonsynonymous mutations across genes using a previously published approach (Good *et al.* 2017). To briefly summarize, gene-level parallelism was assessed by calculating the excess number of nonsynonymous mutations acquired at a given gene, relative to the expectation if mutation accumulation was primarily driven by gene size. This quantity, known as *multiplicity*, is defined as

(5) $$m_{i}\equiv n_{i}\cdot \frac{\bar{L}}{L_{i}},$$

where *n_i_* is the number of mutations observed in the *i*th gene across all 5 replicate populations for a given strain-transfer regime combination. The term *L_i_* is the effective number of nonsynonymous bases in the gene and $\bar{L}$ is the mean number of nonsynonymous bases of all genes. Under this definition, the null hypothesis is that all genes have the same multiplicity $\bar{m}=n_{\mathrm{tot}}/N_{\mathrm{genes}}$. Using the observed and expected values, we quantified the net increase of the log-likelihood of the alternative hypothesis relative to the null

(6) $$\Delta \ell =\sum_{i}n_{i}\;\text{log}\;\left(\frac{m_{i}}{\bar{m}}\right),$$

where significance was assessed using permutation. The null hypothesis that genes acquired a random number of nonsynonymous mutations can be captured through the Poisson distribution (Good *et al.* 2017). Using Equation (5), we defined the single parameter of the Poisson null as $\frac{n_{\mathrm{tot}}L_{i}}{\bar{L}N_{\mathrm{genes}}}$. To identify specific genes that are enriched for mutations, we calculated the *P*-value of each gene using the Poisson distribution as

(7) $$P_{i}=\sum_{n\geq n_{i}}\frac{{\left(\frac{n_{\mathrm{tot}}L_{i}}{\bar{L}N_{\mathrm{genes}}}\right)}^{n}}{n!}e^{-\frac{n_{\mathrm{tot}}L_{i}}{\bar{L}N_{\mathrm{genes}}}},$$

where false discovery rate correction was performed by defining a critical *P*-value ($P^{*}$) based on the survival curve of a null Poisson distribution. To increase statistical power, we only examined a gene within a given strain-transfer regime combination if it accrued at least 3 mutations across all replicate populations $n_{i}\geq 3$.

We then defined the set of significant genes for each strain-transfer combination for α=0.05 as:

(8) $$I=\left\{i:P_{i}\leq P^{*}\left(\alpha \right)\right\}.$$

To determine whether genes associated with the *spo0A* regulon were enriched for nonsynonymous mutations, we used an approach similar to Equation (7). The question of whether a set of genes acquired more mutations than expected by chance can be modeled as a binomial process, where the probability that a given region acquires a mutation is equal its fraction of the genome ($l=L_{\text{region}}/L_{\text{genome}}$). For *spo0A*, $l_{\mathrm{spo}0a}\approx 0.03$. Using this model, we define the following *P*-value.

(9) $$P_{i}=\sum_{n\geq n_{i}}\left(\begin{array}{c} n_{\mathrm{total}}\\ n \end{array}\right)l^{n}{\left(1-l\right)}^{n_{\mathrm{total}}-n}.$$

### (Con/di)vergence at the gene level

We tested for convergent vs divergent evolution by identifying the set of genes that were enriched within a given combination of 2 strain-transfer regimes ($I_{1}\cap I_{2}$). The null distribution for the size of $I_{1}\cap I_{2}$ can be modeled as the process of sampling a given number of genes out of *N_genes_* in the genome without replacement 2 times, a sampling process that is captured by the hypergeometric distribution (Grimmett and Stirzaker 2001). By extending this distribution to the multivariate case (Shoemaker, Polezhaeva *et al.* 2021), we obtained a null probability distribution for the number of genes that are enriched in a given pair of strain-transfer regimes:

$$\begin{array}{c} P(\left|I_{1}\cap I_{2}\right|=k)=\frac{\left(\begin{array}{c} N_{\mathrm{genes}}\\ k \end{array}\right)}{\left(\begin{array}{c} N_{\mathrm{genes}}\\ \left|I_{1}\right| \end{array}\right)\left(\begin{array}{c} N_{\mathrm{genes}}\\ \left|I_{2}\right| \end{array}\right)}\sum_{j=0}^{\text{min}\left\{\left|I_{1}\right|,\left|I_{2}\right|\right\}-k}{\left(-1\right)}^{j}\left(\begin{array}{c} N_{\mathrm{genes}}-k\\ j \end{array}\right)\\ \times \left(\begin{array}{c} N_{\mathrm{genes}}-j-k\\ \left|I_{1}\right|-j-k \end{array}\right)\left(\begin{array}{c} N_{\mathrm{genes}}-j-k\\ \left|I_{2}\right|-j-k \end{array}\right) \end{array}.$$

Using this distribution, we determined whether the number of intersecting enriched genes is greater than (convergence) or less than (divergence) our null expectation.

To examine convergent/divergent evolution among enriched genes, we calculated the vector of relative multiplicities ($M_{i}=m_{i}/\sum m_{i}$) and compared the mean absolute difference between *I* genes for a given pair of transfer regimes or strains as

(10) $$\langle \Delta M\rangle =\frac{1}{I}\sum_{i=1}^{I}\left|M_{i}^{1}-M_{i}^{2}\right|.$$

Null distributions of 〈ΔM〉 were generated by constructing a gene-by-strain mutation count matrix for each transfer regime and randomizing combinations of mutation counts, constrained on the total number of mutations acquired within each gene across strains and the number of mutations acquired within each strain. Randomization was performed for 10,000 iterations using a Python implementation of the ASA159 algorithm. A null distribution was obtained using this approach, from which observed values of 〈ΔM〉 were standardized ($Z_{\langle M\rangle }$) (Patefield 1981; Baak *et al.* 2019). To determine whether spore-forming genes and the *spo0A* regulon were enriched, we calculated the difference in the fraction of nonsynonymous mutations between the WT and Δ*spo0A* strains for a given transfer regime, where the null was obtained using the binomial distribution in Equation (9). Genes in the *spo0A* regulon were identified using a published database (Arrieta-Ortiz *et al.* 2015) and genes associated with endospore formation were identified by annotation. Finally, we identified genes that were preferentially enriched in one background or transfer regime over the other using the Skellam distribution as previously described (Shoemaker and Lennon 2020).

### Wright–Fisher simulations of evolution with a seed bank

To determine whether the empirical patterns of genetic diversity, we observed were consistent with the outcomes predicted by seed bank effects, we performed forward-time Wright–Fisher simulations following a previously described approach (Good *et al.* 2012). Each simulated generation consisted of 3 steps: (1) change of metabolic state, (2), mutation, and (3) reproduction. Dormancy was incorporated in a way similar to previous research efforts (Blath *et al.* 2015, 2016; Blath, Buzzoni, Casanova *et al.* 2019; Blath, Buzzoni, Koskela *et al.* 2019). In a population consisting of *A* active and *D* dormant individuals, we modeled dormancy by considering the number of individuals that entered or exited a dormant state each generation, *c*, chosen so that the number of individuals in a given metabolic state remained constant over time. Given *c*, an active individual enters a dormant state with probability $\frac{c}{A}$ and remains in an active state with probability $1-\frac{c}{A}$. A given dormant individual resuscitates with probability $\frac{c}{D}$ and remains in the seed bank with probability $1-\frac{c}{D}$. The length of time that an individual remains in a dormant state is a geometrically distributed random variable, where the probability of resuscitation (i.e. success probability) is the inverse of the per-individual probability of resuscitation (Table 1). Using this distribution, the impact of dormancy is captured by the mean number of generations that an individual spends in a dormant state, $\langle T_{d}\rangle =D/c$ and the ratio of active and dormant individuals K=A/D.

We focused on the acquisition of de novo mutations among active individuals, as the rate of mutation per-unit time is far higher among individuals that are actively undergoing genome replication (Ryan 1955; Maki 2002; Gangloff *et al.* 2017). Given that the rapid increase in the allele frequencies we could observe was likely predominantly driven by positive selection, we focused on a distribution of fitness effects of beneficial mutations (ρ(s)) that are acquired at a per-individual per-base pair rate of *U_b_*. While the true value of *U_b_* is unknown, we elected for a value that was on the order of magnitude of 10% of the total mutation rate (all nonlethal mutations) obtained from a previously published mutation accumulation experiment $U_{b}=3.28*10^{-11}$ per-individual per-bp (Sung *et al.* 2015). Using this quantity and a known genome size of $L_{\text{genome}}=4.29*10^{6}\text{bp}$, a total of $AU_{b}L_{\text{genome}}$ beneficial mutations were drawn from a Poisson distribution and acquired in the active portion of the population each generation, so that an active individual acquired a beneficial mutation with probability $\frac{AU_{b}L_{\text{genome}}}{A}=U_{b}L_{\text{genome}}$. The fitness effect *s* was drawn from ρ(s) and the fitness of a given lineage *X_i_* was updated at generation *τ* as $X_{i}(\tau )\to X_{i}(\tau )+s$. The form and parameters of ρ(s) are virtually never known a priori and are difficult to infer in evolve-and-resequence evolution experiments (Good and Desai 2015). In light of this fact, we elected to model ρ(s) as an exponential distribution with a scale parameter of $10^{-2}$.

Finally, selection was simulated as a multinomial sampling process. We modeled the selection coefficient of a given individual at a given generation ($s_{i}(\tau )$) as a measure that is relative to the mean fitness of the population at the current generation ($\bar{X}(t)$). After mutations were acquired, the strength of selection on the *i*th individual was calculated as $s_{i}(\tau )=X_{i}(\tau )-\bar{X}(\tau )$. Then, *A* individuals were drawn from a multinomial distribution after the mutation step, where the *i*th individual was sampled with probability $e^{s_{i}(\tau )}/\sum_{j}^{A}e^{s_{j}(\tau )}$. Simulations were run for 3,300 generations with $A=10^{6}$ for $c=10^{-5}$ and values of *D* ranging from $10^{1}-10^{6}$. Only values of *D* were manipulated as the same transition rates can be obtained by manipulating *c*. Ten replicate simulations were performed for each value of *D*. All simulations are in the Python script run_simulations.py.

## Results and discussion

**Fig. 1.**
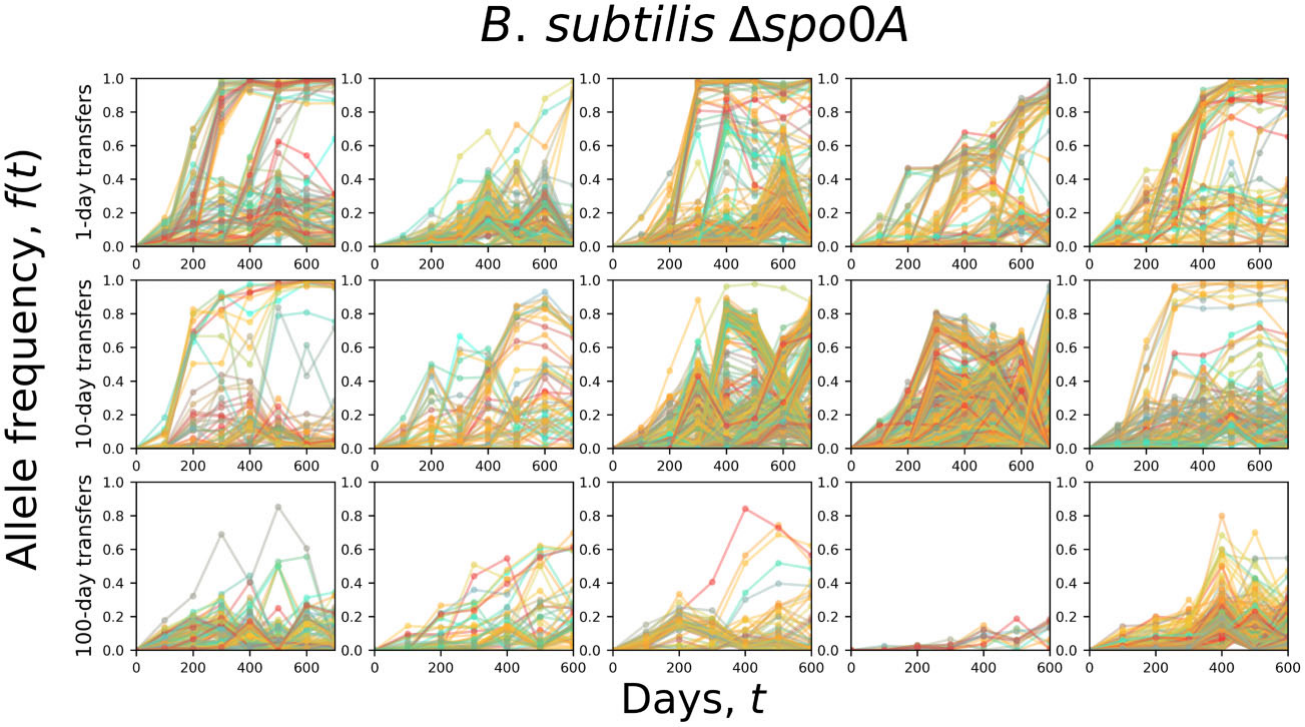
The allele frequency trajectories of *B. subtilis* populations that were unable to form seed banks (Δ*spo0A*) exhibited qualitatively different dynamics across transfer regimes. Typically, a lower number of mutations and fixation events accumulated in the 10-day regime relative to the 1-day regime. There were even fewer detectable mutations in the 100-day regime, a likely consequence of the low number of generations that occurred over 700 days. All Δ*spo0A* replicate populations are included on this plot.

**Fig. 2.**
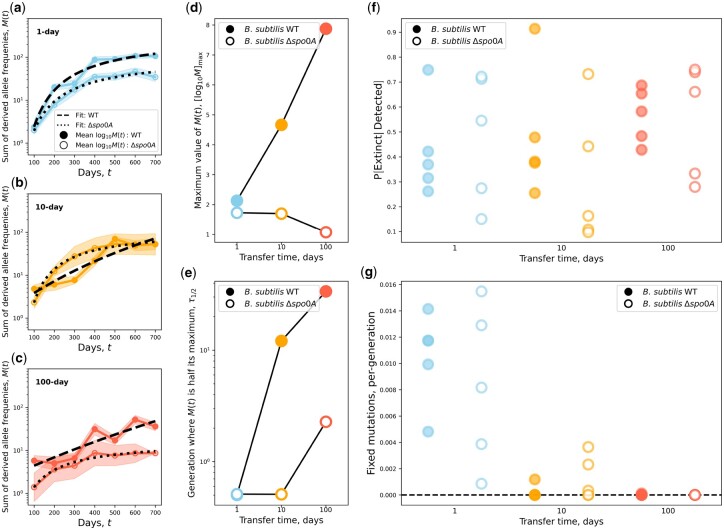
The presence of a seed bank altered the accumulation of genetic diversity. a–c) By examining the sum of derived allele frequencies (*M*(*t*), Equation 2), we were able to summarize the accumulation of de novo mutations over time for all strains and transfer regimes. The WT strain had higher a mean ${\;\text{log}\;}_{10}M(t)$ than Δ*spo0A* and the relationship between *t* and ${\;\text{log}\;}_{10}M(t)$ became noticeably more linear as transfer time increased for the WT strain. Shaded areas represent the standard error of mean ${\;\text{log}\;}_{10}M(t)$. d, e) To quantify the effect of seed bank formation on this empirical relationship, we formulated a phenomenological model that allowed us to summarize the curve through 2 parameters: the maximum amount of genetic diversity that could accumulate (${\left[{\;\text{log}\;}_{10}M\right]}_{\text{max}}$) and the number of generations until half of the maximum is reached ($\tau_{1/2}$; Equation 3). Values of ${\left[{\;\text{log}\;}_{10}M\right]}_{\text{max}}$ for the WT strain steadily increased with transfer time while Δ*spo0A* remained consistent with the prediction that the presence of a seed bank increases the amount of genetic diversity that a population can maintain. Conversely, $\tau_{1/2}$ decreased for Δ*spo0A* but remained constant for the WT, consistent with the prediction that the rate of molecular evolution would increase in the absence of a seed bank. f, g) However, the effect of seed banks on the final states of alleles was less straightforward. While fixation events occurred across transfer regimes and strains, there was substantial variation across replicate populations that made it difficult to determine whether the presence of a seed bank affected the probability of fixation or the rate of molecular evolution (per-generation number of substitutions).

**Fig. 3.**
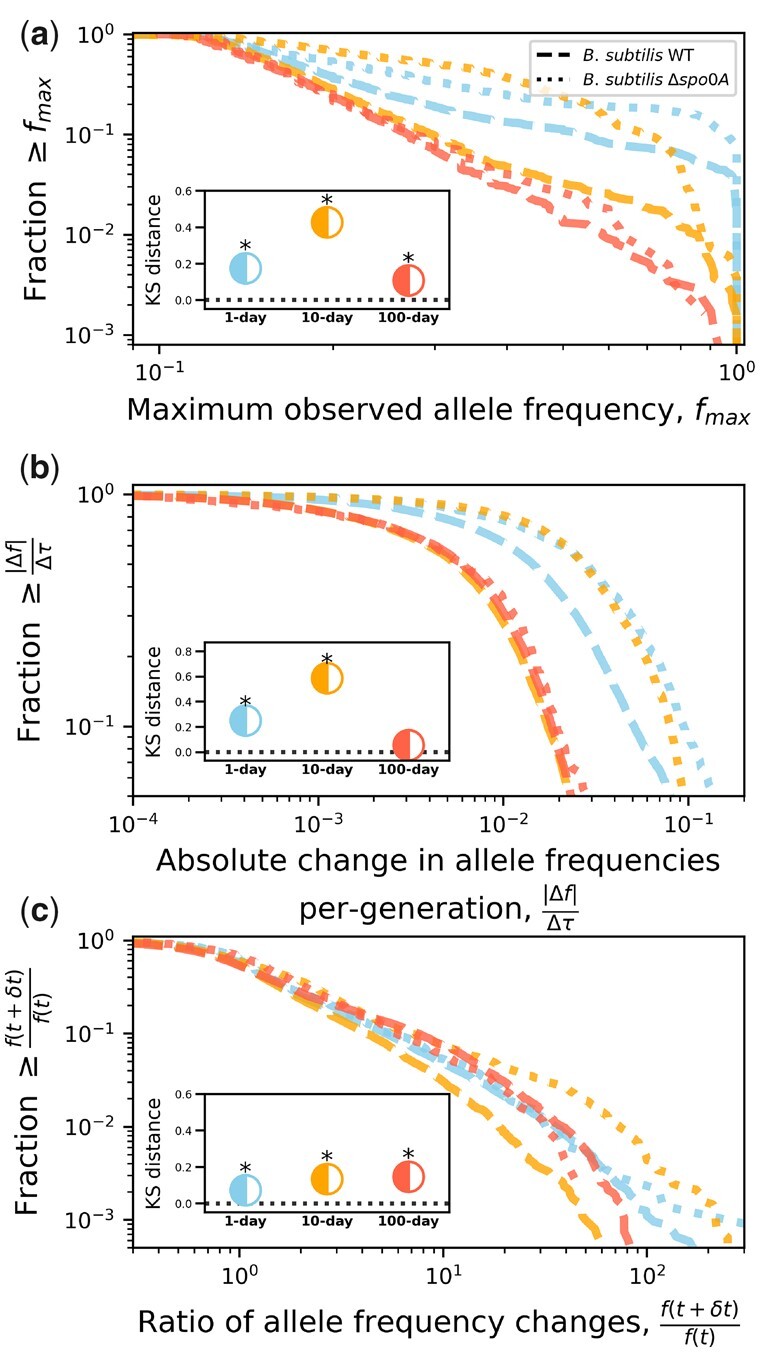
Due to the low number of fixation events, we devised alternative measures of molecular evolution to evaluate the effect of seed banks. We examined 3 measures: (a) the maximum frequency realized by an allele, (b) the per-generation magnitude of change in allele frequency, and (c) the change in the direction of allele frequencies between time points (Equation 4). These measures were examined by calculating the empirical survival distribution (the complement of the empirical cumulative distribution function) for a given strain-transfer combination. The typical value of all 3 measures was higher for Δ*spo0a* than the WT across transfer regimes, consistent with the predicted effect of a seed bank. The difference we observed between strains was confirmed via KS tests for all transfer regimes (Benjamini–Hochberg corrected *P*-values <0.05 marked by an asterisk).

**Fig. 4.**
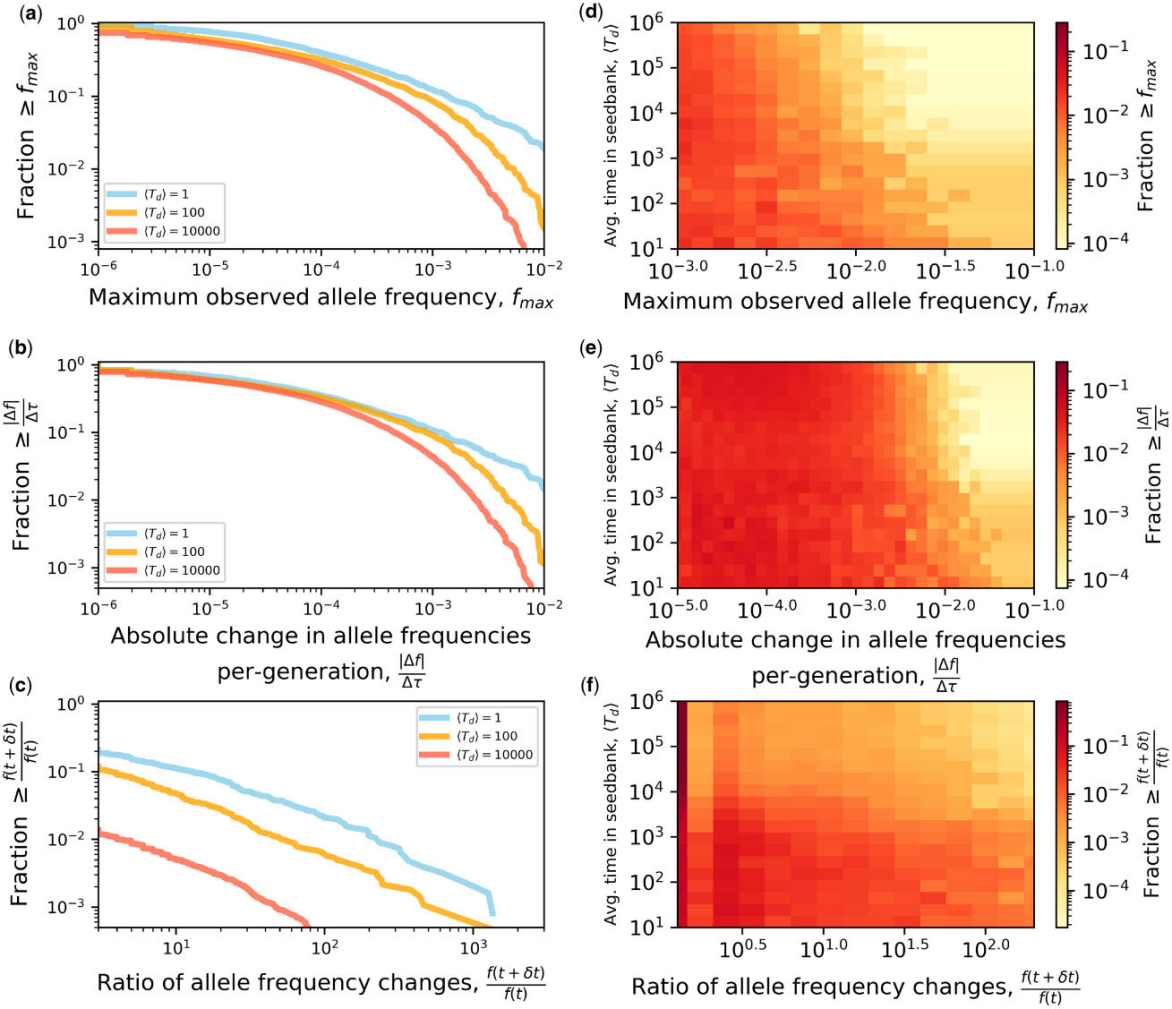
To validate our empirical observations, we performed forward-time population genetic simulations of populations with seed banks of varying size, where the effect of seed banks can be summarized by the average number of generations that an individual spends in a dormant state ($\langle T_{d}\rangle$; *Materials and Methods*). a-c) The breadth of simulated survival distributions gradually decreased with $\langle T_{d}\rangle$ for all 3 measures of allele frequencies (Equation 4), recapitulating the empirical results described in Fig. 3. d-f) The robustness of this pattern is made evident by performing simulations across a wide range of $\langle T_{d}\rangle$ values.

**Fig. 5.**
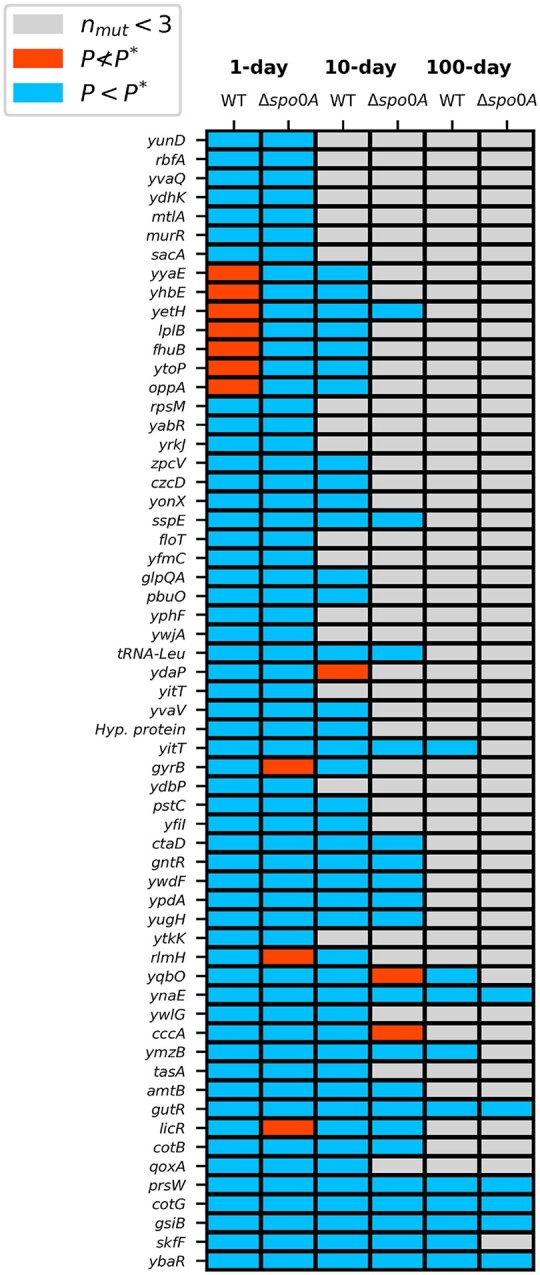
By comparing the set of genes that contributed toward parallel evolution with a given strain-transfer combination, we visualize patterns of convergent and divergent molecular evolution. While all strain-transfer combinations consistently acquired more nonsynonymous mutations than expected by chance at a large number of genes ($P<P^{*}$), very few genes were enriched exclusively within a given strain. Those genes that were significantly enriched within a given strain combination typically also acquired mutations at a nonsignificant level ($P≮P^{*}$) in the remaining strain, suggesting that the removal of endospore formation did not generate evolutionary trajectories that were divergent in terms of gene identity. To increase statistical power, we ignored all genes that acquired less than 3 mutations (*n_mut_* < 3) across all 5 replicate populations for a given strain-transfer regime combination. Gene names are listed as provided in the annotated reference genome; all other names were acquired using RefSeq IDs (see Supplementary File 1 for gene metadata).

**Fig. 6.**
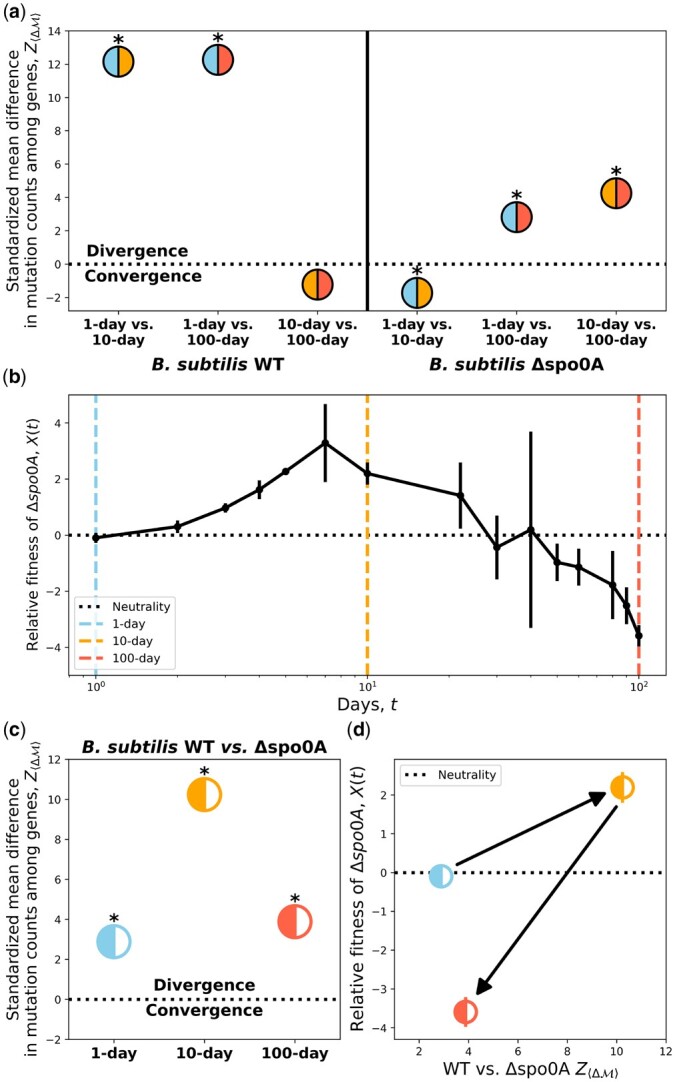
By examining the number of mutations within each gene, we determined whether convergent or divergent evolution occurred between a given pair of transfer regimes or strains by calculating the mean absolute difference of mutation counts across genes (〈ΔM〉; Equation 10). a) A comparison between all transfer regime combinations within each strain reveals contrasting dynamics of divergence/convergence. Divergent evolution initially occurred among the WT background initially for 1- vs 10-day and 1- vs 100-day comparisons, with 10- and 100-day transfers having a weak signal of convergence. This pattern became inverted for Δ*spo0A*, as 1- and 10-day transfers converged while the remaining transfer regime combinations diverged. For comparisons between strains, we compared signals of convergent/divergent evolution between WT and Δ*spo0A* strains with the transfer regime-dependent fitness effects of removing *spo0A*. b) A 100-day fitness assay between Δ*spo0A* and WT strains revealed the time-dependent fitness effect of the ability to form endospores. In the 1-day regime, there is no fitness effect of *spo0A* removal, which ultimately becomes beneficial by day 10. However, shortly thereafter it became highly deleterious. Black dots represent the mean of 3 replicate assays while bars represent the standard error. c) Divergent evolution consistently occurred across transfer regimes, with the 10-day transfers harboring the strongest signal of divergent evolution. d) Mapping signals of divergent evolution to estimate fitness, we determined how the sign and magnitude of selection changed with the degree of divergent evolution. Asterisks denote *P < *0.05.

By reconstructing the molecular fossil record of our experiment, we examined the trajectories of de novo mutations for all replicate populations (Fig. 1). The molecular evolutionary dynamics of *B. subtilis* largely followed our predictions, as the presence of a seed bank increased the maximum amount of genetic diversity retained by a population and decreased its per-generation rate of accumulation (Fig. 2; Table 2). Measures that capture distinct features of mutation trajectories were also largely consistent with our predictions (Fig. 3), results that were validated with forward-time population genetic simulations (Fig. 4). By comparing gene mutation counts between strain-transfer combinations (Figs. 5 and 6; Table 3), we determined that the ability to enter a dormant state radically altered the targets of molecular evolution, though the strength and direction of the effect were environment-dependent. The results of this long-term experiment test, confirm, and challenge long-standing hypotheses regarding the effect of seed banks on the dynamics of molecular evolution.

**Table 3.** The number of genes that were enriched for nonsynonymous mutation solely within a given strain-transfer regime combination (i.e. unique genes).

Number unique enriched genes		
Transfer regime	*B. subtilis* WT	*B. subtilis* Δ*spo0A*
1 day	77	2
10 days	12	8
100 days	12	0

### The demographic effect of seed banks in rapidly evolving populations

While Δ*spo0A* populations were unable to form endospores, the fraction of endospores varied across transfer regimes for WT lines, providing the mean to assess the impact of seed bank formation on molecular evolutionary dynamics. Endospores were not detected in the 1-day transfer regime for the WT; though by the end of 10 days, the proportion of spores reached 0.65 ± 0.062 (mean ± standard error). This estimate of the fraction of spores was consistent with prior energy-limitation experiments with *B. subtilis* (Shoemaker, Jones *et al.* 2021).

Despite the inability to form protective structures, all replicate populations of Δ*spo0A* from all transfer regimes survived the experiment. This pattern of consistent demographic survival is a marked difference from a previous experiment that used an identical energy-limitation design, where bacterial taxa from diverse phyla (Bacteroidetes, Proteobacteria, and Deinococcus-Thermus) frequently underwent demographic extinction (i.e. *N *=* *0) in 10- and 100-day transfer regimes (Shoemaker, Polezhaeva *et al.* 2021), whereas *Bacillus* WT never went extinct. This comparative result suggests that *Bacillus* is exceptionally capable of surviving and evolving in harsh environments, even without access to what is generally considered its primary survival strategy of endospore formation.

### Seed banks altered the accumulation, but not the fates, of de novo mutations

Through pooled population sequencing, we reconstructed the trajectories of de novo mutations for all 5 replicate populations from each transfer regimes (Fig. 1; Supplementary Fig. 2). By examining the sum of derived allele frequencies at a point in time (*M*(*t*)), we were able to examine how de novo mutations accumulated over the course of the experiment for all replicate populations (Fig. 2, a–c, Supplementary Fig. 3). Populations capable of forming seed banks tended to have higher value of *M*(*t*) in the 1 and 100-day transfer regimes, though this difference is not as apparent for the 10-day regime.

These patterns spurred the development of a novel mathematical model. Intuitively, seed banks can affect the accumulation of de novo genetic diversity over time through 2 means: (1) increasing the maximum amount of diversity that a populating can retain and (2) reducing the rate that diversity accumulates. Building off of this assessment, we developed a phenomenological model with 2 intuitive parameters (Equation 3) that can be used to test our predictions, the maximum value of the logarithm of $M(t)\;\left({\left[{\;\text{log}\;}_{10}M\right]}_{\text{max}}\right)$ and the number of generations required for ${\;\text{log}\;}_{10}M$ to reach half of its maximum value ($\tau_{1/2}$). Estimates of these parameters obtained through numerical optimization and estimates of the number of generations that accrued within each treatment (Table 2) revealed that ${\left[{\;\text{log}\;}_{10}M\right]}_{\text{max}}$ remained fairly constant as energy-limitation (i.e. time between transfers) increased for the WT, but sharply decreased for Δ*spo0A* (Fig. 2d). In contrast, $\tau_{1/2}$ remained constant for Δ*spo0A*, but decreased as transfer time increased (Fig. 2e). This pattern was consistent with our prediction that the rate of accumulation of genetic diversity will be higher in populations that cannot form a seed bank.

While the trends we observed were generally robust, the error in our estimates for the 100-day transfer regime was considerable (Supplementary Table 2). This error was likely a result of the small number of mutations acquired among populations in the 100-day treatment as well as their small population sizes, increasing the variance in our estimates of *M*(*t*). Regardless, parameter estimates consistently change in directions predicted by the seed bank effect.

Given that seed banks altered the accumulation of genetic diversity, we then examined how the presence of a seed bank altered the fate of a given mutation. The presence of seed banks reduced the efficiency of selection and retain genetic diversity (Schoen *et al.* 1998), suggesting that a given mutation would have a lower probability of extinction as well as a lower substitution rate (Blath *et al.* 2015; Koopmann *et al.* 2017). Our estimates of the probability of extinction for a given mutation provide little evidence to support this claim, as there was substantial variation across replicate populations for a given strain-transfer combination (Fig. 2f). This result suggests that seed banks primarily altered the accumulation of genetic diversity by buffering the dynamics of segregating mutations rather than reducing their rate of extinction. However, we note that this study, as is typical of microbial pooled sequencing studies, has a lower frequency resolution $\;O(10^{-2})$. Given the large empirical sizes of our experimental populations, we were unable to evaluate the effect of dormancy on the evolutionary fates of mutations across a wide range of frequency values ($1/N<f≲10^{-2}$).

While the rate of fixation was similar for WT and Δ*spo0A* in the 1-day regime, as expected given that the ability to form a seed bank does not contribute to survival on that time scale, few fixation events occurred within replicate populations in the 10- and 100-day transfer regimes (Fig. 2g). This paucity of fixations meant that the substitution rate could not be estimated for certain transfer regimes, preventing comparisons from being performed. While the strength and direction of selection could be evaluated by examining the proportion of nonsynonymous to synonymous mutations (*pN*/*pS*), estimates of *pN*/*pS* were consistently less than one across transfer regimes and there was no difference in *pN*/*pS* values between WT and Δ*spo0A* populations within a given transfer regime (Supplementary Fig. 4). This lack of difference suggests that purifying selection was predominant regardless of whether a population could enter the seed bank or in environments where the ability to enter a dormant state would be favorable. This consistent lack of difference is unlikely to be the result of our choice of knockout gene, as genome-wide signals of *pN*/*pS* were not affected by the exclusion of genes in the *spo0A* regulon (Supplementary Table 3).

### Seed banks altered the dynamics of segregating mutations

While seed banks had a clear effect on the accumulation of de novo genetic diversity, they did not substantially alter the evolutionary fates of mutations. In addition, few if any fixation events were inferred for 10- and 100-day transfer regimes. These 2 results indicate that the substitution rate and the probability of extinction of a mutation are uninformative for this particular study. Such quantities are useful in that their value in the presence of a seed bank can be compared to predictions from existing mathematical models (Blath *et al.* 2015, 2016; Koopmann *et al.* 2017). However, their emphasis on the final state of a given mutation (i.e. fixation or extinction) means that these quantities do not necessarily capture the dynamics that mutations exhibit over their sojourn times. Therefore, we leveraged the temporal structure of the data by examining mutation trajectories in order to evaluate the extent that seed banks alter the dynamics of molecular evolution.

We examined 3 measures of each mutation trajectory: (1) the maximum frequency that a mutation reached (*f_max_*), (2) the set of per-generation magnitudes of frequency changes ($\frac{\left|\Delta f\right|}{\Delta \tau }$) over time, and (3) the set of changes in the direction of allele frequency changes between observations over time (f(τ+Δτ)/f(τ)). By calculating empirical survival distributions for a given measure for each strain-treatment combination, we determined the degree that seed banks altered the molecular evolutionary dynamics of *B. subtilis*.

First, given the dearth of fixation events, it is worth investigating whether the maximum frequency that a mutation obtained over its sojourn time (*f_max_*) is a sufficient proxy of fixation. The condition that a mutation reached a sufficiently high-frequency guarantees fixation under a single-locus model of evolution (Ewens 2004). Though this certainty is not necessarily the case when multiple beneficial mutations simultaneously segregate, where recombination, defined here as the exchange of genetic material between organisms, may not be sufficiently high to justify the assumption of quasi-linkage equilibrium (i.e. independent evolution among multiple sites). Regardless, there are various derivations in theoretical population genetics indicating that *f_max_* is reflective of the strength and direction of selection as well as the conditional probability of fixation of an allele (Fisher 2007; Cvijović *et al.* 2018; Good 2020).

We do not infer fixation events in several replicate populations, so we cannot confirm that there is a positive empirical relationship between *f_max_* and the probability of fixation. However, we inferred a large number of mutation extinction events across all replicate populations. Given that a segregating allele has to eventually become extinct of fixed, the probability of fixation can be viewed as the complement of the probability of extinction (Pr[fixation=1−Pr[extinction]), meaning that if *f_max_* is reflective of fixation then we should observe an inverse relationship between estimates of Pr[extinction|detected] and *f_max_*. We find that this prediction holds, as all strain-transfer regime combinations exhibited the inverse relationship (Supplementary Fig. 5), allowing us to proceed with our analyses of the distribution of *f_max_*.

Across transfer regimes, Δ*spo0A* populations had higher values of *f_max_* than their corresponding WT transfer regime and the significance of the distance between the distributions was tested and confirmed via KS tests (Fig. 3a). Interestingly, WT and Δ*spo0A* populations had the smallest distance in the 100-day transfer regimes, which is likely due to the comparatively brief evolutionary timescale reducing the maximum attainable frequency of a de novo mutation. Overall, the values of *f_max_* exhibited in the presence and absence of a seed bank are consistent with the prediction that the presence of a seed bank will reduce the rate of molecular evolution.

The presence of a seed bank generated a similar effect on the magnitude of allele frequency changes between observations ($\frac{\left|\Delta f\right|}{\Delta \tau }$) and the direction of frequency changes (f(τ+Δτ)/f(τ); Fig. 3, b and c). There was a small, but significant difference where Δ*spo0A* had a higher magnitude of change. This distance increased considerably for the 10-day transfer regime, consistent with the prediction that the presence of a seed bank would buffer temporal changes in allele frequencies. Though much of this distance disappeared for the 100-day transfer regime, again, a likely result of the comparatively brief evolutionary timescale of the treatment.

While the distributions of quantities calculated from mutation trajectories match our predictions regarding the effect of seed banks on molecular evolutionary dynamics, they are population genetic quantities that are not typically examined. Arguably, this reflects the historic difficulty of obtaining temporally resolved frequency trajectories for a large number of mutations, rather than the measures themselves being uninformative. To evaluate how these measures of mutation trajectories typically behave in the presence of a seed bank, we simulated the evolutionary dynamics of an adapting population with seed bank dynamics as a Wright–Fisher process (Blath *et al.* 2015, 2016) (Equation 4). Briefly, in a population with *A* active individuals *c* individuals enter a seed bank of size *D*, while a corresponding number of individuals are resuscitated from their dormant state. The effect of this dynamic can be captured by the average number of generations that an individual spends in a dormant state $\left(\langle T_{d}\rangle =D/c\right)$ and the ratio of active and dormant individuals K=A/D (Blath *et al.* 2015). While *c* is difficult to estimate, *A* and *D* can readily be measured, providing empirical constraint on the range of parameter values in our simulations. Furthermore, endospores are undetectable in WT lines and absent entirely in Δ*spo0A* (i.e. *D *=* *0), meaning that the difference between WT and Δ*spo0A* mutation trajectory statistics in Fig. 3 for 1- and 10-day regimes can be viewed as the degree that the ability to enter a dormant state decreased the typical value of a given mutation trajectory statistic. We captured this seed bank effect by performing simulations across a range of values of *c* for empirically informed values of *A* and *D* and estimating the measures in Equation (4) from simulated trajectories. Using this approach, we were able to recapitulate our empirical survival distributions (Fig. 4). These principled simulations combined with empirical observations confirm long-standing untested hypotheses regarding the effect of seed banks dynamics of molecular evolution.

The Δ*spo0A* populations were incapable of forming endospores and exhibited mutation trajectories that were consistent with an absence of a seed bank. However, *spo0A* can regulate the genetic competence of *B. subtilis* (Albano *et al.* 1987; Hahn *et al.* 1995; Schultz *et al.* 2009), which in turn affects the rate of recombination. While the rate of recombination has not been directly estimated in *Bacillus*, population genetic inference procedures suggest that the ratio of recombination relative to mutation is ≈1 (Vos and Didelot 2009). Given that within the genus the rate of recombination is estimated to be on the same order of magnitude as that of mutation, a major reduction in the rate of recombination due to the removal of *spo0A* could greatly alter the molecular evolutionary dynamics of *Bacillus* (Dixit *et al.* 2017). To determine whether Δ*spo0A* populations had a lower recombination rate, we calculated the squared correlation coefficient for all pairs of segregating mutations within each replicate population. This measure has previously been used to examine the effect of recombination on rapidly evolving microbial populations (McDonald *et al.* 2016). By pooling coefficients across replicate populations and comparing the distribution of coefficients of the WT and Δ*spo0A* strains, we found that the degree of correlation was consistently greater for Δ*spo0A* across transfer regimes (Supplementary Fig. 6a). This result suggests that the rate of recombination was lower in Δ*spo0A* populations relative to the WT. To determine whether this pattern was driven by the absence of a seed bank, we calculated distributions of correlation coefficients from simulated data obtained using Equation (4) and found that seed banks alone were insufficient to cause the patterns we observed (Supplementary Fig. 6b).

While the distribution of correlation coefficients suggests that the rate of recombination was lower in Δ*spo0A* populations, this does not prove that the statistical patterns we observed in Fig. 3 were caused by a difference in recombination rates between Δ*spo0A* and the WT. To determine whether recombination could feasibly be responsible for the distribution of single-locus measures we examined, we developed and implemented a novel simulation based on recent theoretical developments where 2 alleles on different loci segregate in a population with dormant and active individuals (Supplementary Information; Equation 1) (Good 2020). We found no evidence that the KS distance between dormancy-capable and incapable populations was dependent on the population scaled recombination rate for all trajectory summary statistics (Supplementary Fig. 7), suggesting that any purported difference in the recombination rate between WT and Δ*spo0A* would not be capable of generating the between-strain KS distance values estimated in the 10-day transfer regime. Thus, the analyses presented in Fig. 3 reflect the presence of a seed bank.

It is necessary to consider the possibility that demographic fluctuations due to changing population size could have contributed to the patterns we observed. In a strict sense, the term “bottleneck” does not accurately capture the demographic dynamics of this experiment, as populations did not exhibit a near-instantaneous decline on a logarithmic scale (Supplementary Fig. 8). It is also becoming increasingly apparent that while the net rate of growth (birth minus death) remains negative over time in microbial populations maintained in flasks over an extended period of time without resource replenishment (i.e. 10- and 100-day transfer regimes), the net rate of growth rate tends to increase with time (Ryan 1959; Finkel and Kolter 1999; Avrani *et al.* 2017; Shoemaker, Jones *et al.* 2021). This consistent demographic pattern of an increasing net rate of growth is antithetical to the interpretation of a population bottleneck.

However, we can glean qualitative insight into the extent that demographic fluctuations are capable of explaining the patterns we observe. There is an inconsistent pattern in the estimates of the ratio of the final and initial size of the population ($\frac{N_{f}}{N_{i}}$) across transfer regimes between the WT and Δ*spo0A*, a statistic that captures the degree of demographic fluctuations between transfers. The ratio $\frac{N_{f}}{N_{i}}$ for the WT is higher than that of Δ*spo0A* in the 10-day transfer regime by roughly an order of magnitude. However, this pattern is reversed in the 100-day transfer regime, as $\frac{N_{f}}{N_{i}}$ is higher for Δ*spo0A* (Table 2). This inconsistency contrasts with the consistent trends we observe for ${\left[{\;\text{log}\;}_{10}M\right]}_{\text{max}}$ and $\tau_{1/2}$, where the WT estimates of both quantities are higher than those of Δ*spo0A* for both 10- and 100-day transfer regimes (Fig. 2, d and e). Given these consistent patterns, if demographic fluctuations were the principal contributor toward the accumulation of de novo genetic diversity, we would expect $\frac{N_{f}}{N_{i}}$ to be greater for a single strain across all transfer regimes.

Finally, it is necessary to consider how fluctuations in population size could contribute to the mutation trajectories we observe. A large reduction in the size of a population will typically increase the magnitude of fluctuations in the frequency of an allele (Ewens 2004). In the context of this study, we examined the fluctuation of allele frequencies by calculating the per-generation absolute difference in allele frequencies between consecutive time points ($\frac{\left|\Delta f\right|}{\Delta \tau }$; Equation 4b). By examining this distribution, we found that the KS distance between the WT and Δ*spo0A* strains was at its largest for the 10-day treatment, with a typical value of $\frac{\left|\Delta f\right|}{\Delta \tau }$ for Δ*spo0A* larger than the WT by roughly an order of magnitude. This result contrasts with our expectations based solely on population size, where we would expect that the treatment with the larger reduction in population size (i.e. the WT) would exhibit larger allele frequency fluctuations. While there was no significant difference in the 100-day transfer regime, the typical value of $\frac{\left|\Delta f\right|}{\Delta \tau }$ for Δ*spo0A* decreased despite the fact that its value of $\frac{N_{f}}{N_{i}}$ was ∼70 times larger than that of the WT. This result, again, is inconsistent with what we would expect if fluctuations in population size were a major contributing factor.

### Parallelism and (con/di)vergence at the gene level

To determine whether endospore formation as a life-history strategy affects the targets of molecular evolution in addition to its direction, it was necessary to first identify the potential contributors of adaptation among replicate populations within a given strain-transfer combination. To identify potential contributors toward adaptation, we examined the distribution of nonsynonymous mutation counts across genes within each strain-transfer combination. The log-likelihood that some number of genes were enriched for nonsynonymous mutations (Δℓ) was significant across 1-, 10-, and 100-day transfer regimes for both strains (Supplementary Table 4). However, values of Δℓ tended to be slightly elevated for the WT strain, suggesting that a higher degree of parallel evolution occurred when endospore formation was possible. To determine whether this difference between strains was real or an artifact of the higher number of mutations acquired by the WT strain across transfer regimes, we randomly subsampled mutations to obtain a distribution of Δℓ values for each strain-transfer combination. While the difference in Δℓ values between strains was greatly reduced, the WT strain consistently had a higher degree of genome-wide parallelism across transfer regimes (Supplementary Fig. 9a). This increased degree of parallelism suggests that the presence of a seed bank can make the molecular targets of evolution more predictable. However, it is worth considering whether this result is due to the use of Δ*spo0A*. The gene *spo0A* is a master regulator that controls cellular processes in addition to endospore formation (Hoch 1971; Dubnau 1991), meaning that the decrease in parallelism in Δ*spo0A* could be a consequence of pleiotropy caused by the *spo0A* regulon. That the *spo0A* regulon is enriched for nonsynonymous mutations in the 1-day transfer regime (Supplementary Table 5) supports this hypothesis. To investigate this possibility, we performed the same subsampling procedure on the set of genes that were not in the *spo0A* regulon and compared the subsampled likelihood to the result obtained by using all genes in the genome. We found the contribution of *spo0A* to the genome-wide signal of parallelism to be inconsequential, suggesting that the deletion of *spo0A* was not responsible for the patterns of parallel evolution we observed (Supplementary Fig. 9b).

To deconstruct this genome-wide pattern of parallelism, we examined the excess number of nonsynonymous mutations at a given gene (i.e. multiplicity; Good *et al.* 2017). As expected, the distribution of multiplicities of the WT was consistently higher than Δ*spo0A* across transfer regimes (Supplementary Fig. 10, a–c), suggesting that this effect is independent of energy limitation and is instead likely driven by the larger number of nonsynonymous mutations that accrued in the WT strain. To account for this difference, we calculated a *P*-value for each gene within each strain-transfer combination, allowing us to pare down the total number of genes in the genome to a small number of genes that were disproportionately enriched for nonsynonymous mutations, the putative targets of selection. Identifying the set of significantly enriched genes revealed that genes enriched within the WT for a given transfer regime also tended to be enriched within Δ*spo0A* (Fig. 5, Supplementary Fig. 10, d–f). This pattern of consistent enrichment occurred across transfer regimes as well as among transfer regime comparisons within a given strain (Supplementary Figs. 11 and 12), suggesting that, generally, the direction of evolution at the gene level tended toward convergence rather than divergence. We found that this is the case, as the degree of overlap in enriched genes relative to a null distribution suggests convergent evolution (Supplementary Fig. 13; Shoemaker, Polezhaeva *et al.* 2021). While certain transfer regime and strain comparisons had stronger signals of convergence than others, overall convergent evolution overwhelmingly occurred.

Similar to a previous analysis (Shoemaker, Polezhaeva *et al.* 2021), it is likely that gene identity was, again, too coarse a measure to determine whether convergent or divergent evolution occurred. While Δ*spo0A* is a master regulatory gene, its removal may have only slightly perturbed the rates of evolution across a large number of genes in a given environment. If true, then it is arguably more appropriate to examine the difference in mutation counts among enriched genes in order to assess whether convergent or divergent evolution occurred. By examining the mean absolute difference in mutation counts across enriched genes between 2 transfer regimes (〈ΔM〉) and standardizing the observed value with respect to a null distribution ($Z_{\langle \Delta M\rangle }$) obtained via permutation, we established whether convergent or divergent evolution occurred. The WT strain exhibited significant divergent evolution for the 1- vs 10-day and 1-day vs 100-day comparisons, a result that was consistent with the WT surviving energy-poor environments by forming endospores as a life-history strategy (Fig. 6a). This conclusion is strengthened by the evidence of convergent evolution for the 10- vs 100-day comparison, though it was ultimately not significant. For Δ*spo0A* the pattern changed in a manner that resembled a reflection of the WT pattern. There was a significant signal of convergent evolution for the 1- vs 10-day comparison though for the 1- vs 100-day and 10- vs 100-day comparisons we found a strong signal of divergent evolution. To summarize, the removal of Δ*spo0A* generated opposite trends in the direction of molecular evolution at the gene level across combinations of transfer regimes.

To examine how seed bank formation affected the direction of molecular evolution at the gene level within a given transfer regime, we repeated the convergent/divergent analysis between the WT and Δ*spo0A* populations, analyses that can be bolstered through comparisons to fitness estimates (Fig. 6b). Divergent evolution overwhelmingly occurred for all 3 comparisons, though it was at its highest for the 10-day transfer regime (Fig. 6c). This increased divergence for the 10-day transfer regime, along with evidence of convergent evolution for the 1- vs 10-day comparison, may reflect adaptation of Δ*spo0A* to this specific regime. Though, again, it is worth investigating the contribution of genes within the *spo0A* regulon, as the fraction of nonsynonymous mutations in the *spo0A* regulon was significantly higher in the Δ*spo0A* lines relative to the WT for the 10- and 100-day transfer regime (Supplementary Table 6). However, it is unlikely that the pleiotropic nature of *spo0A* was responsible for the differing patterns of convergence and divergence observed between WT and Δ*spo0A* strains, as we obtained virtually indistinguishable estimates of $Z_{\langle \Delta M\rangle }$ when genes in the *spo0A* regulon were excluded from our analysis (Supplementary Fig. 14).

Our estimates of 〈ΔM〉 between strains for a given transfer regime can be compared to the corresponding fitness effect of Δ*spo0A*, allowing us to examine how the sign and magnitude of fitness changes with the direction of molecular evolution. We found that after 1 day of coculture that the fitness difference of Δ*spo0A* relative to the WT was undetectable via CFU counts, meaning that the fitness effect of being unable to form endospores was effectively zero. This result was consistent with the hypothesis that endospore formation has a negligible fitness effect in environments where it is unlikely to be expressed and, thus, would not be advantageous. However, we note that the removal of *spo0A* could have fitness effects in different environments that vary in other aspects but retain the regular resource replenishment of our experiment.

We found that the increase in fitness of Δ*spo0A* for the 10-day transfer regime was accompanied by an increase in the degree of divergent evolution (Fig. 6d). This result suggests that the ability to form endospores actually conferred a temporary fitness disadvantage at the 10-day mark. However, by day 100, the fitness benefit of Δ*spo0A* had dissipated and the magnitude of divergent evolution had diminished, suggesting that it is unlikely that Δ*spo0A* was able to adapt to the harsh 100-day environment. An analysis of the set of genes that were enriched for mutations within a specific strain-transfer combination supports this conclusion (Shoemaker and Lennon 2020). The 100-day Δ*spo0A* was the only strain-transfer combination with no unique enriched genes (Table 3; Supplementary File 2), suggesting that Δ*spo0A* may have been unable to adapt to this extremely energy-limited environment.

While it is unlikely that Δ*spo0A* was able to adapt to the 100-day transfer regime, conversely, the 10-day Δ*spo0A* transfer regime harbored the highest number of unique enriched genes for all 3 Δ*spo0A* transfer regimes, suggesting that adaptation may have occurred even in the absence of endospore formation. The mechanism responsible for the temporary gain in fitness of Δ*spo0A* is unknown, though it is likely partially due to the recycling of dead cells, a phenotype that allows individuals to exploit an untapped resource (Rozen *et al.* 2009; Bradley *et al.* 2018; Shoemaker, Jones *et al.* 2021). Naturally, dormant cells cannot use this resource as their metabolism is effectively nonexistent and, in the case of endospores, metabolically inert, leaving Δ*spo0A* with unrestricted access. Regardless, for the purposes of this study, the removal of endospore formation as a life-history strategy provided a clear fitness benefit in certain energy-limited environments.

The disuse of a trait frequently results in a relaxation of selective pressure on its underlying loci (Maughan *et al.* 2006, 2007). Given that endospore formation did not occur in the 1-day transfer regime, it is possible that the pathways encoding said life-history trait were susceptible to decay due to relaxed selective pressure. By calculating the fraction of nonsynonymous mutations in genes that encode for endospore formation and calculating the difference between Δ*spo0A* and the WT, we found that endospore encoding genes were slightly enriched in the WT for all transfer regimes, a difference that was significant using null distributions simulated via binomial sampling (Supplementary Table 7). Operating under the premise that the majority of endospore-forming genes are nonfunctional in Δ*spo0A* populations, for WT populations in the daily transfer regime this result can be viewed as the outcome of positive selection for the removal of endospore formation as an energetically costly trait in energy-replete environments (Bradshaw *et al.* 1998; Cáceres and Tessier 2004). Prior studies, as well as the spore accumulation assays we performed, support this conclusion (Maughan *et al.* 2007, 2006, 2009). The ability to form endospores became rapidly diminished for populations in the 1-day transfer regime over the first 500 days of the experiment, to the point that it took 10 days for 10% of the population to form endospores (Supplementary Fig. 15). However, the question remains as to why 100-day WT populations acquired a greater fraction of nonsynonymous mutations in endospore encoding genes. Endospore formation underwent no noticeable decline in the 100-day regime (Supplementary Fig. 15), suggesting that these mutations had negligible or even positive effects.

### Conclusion

We demonstrated that the ability to form seed banks altered the molecular evolutionary dynamics of microbial populations. Populations capable of forming seed banks consistently accumulated higher levels of genetic diversity and had a reduced rate of molecular evolution. Through forward-time simulations, we were able to recapitulate empirical observations on the effect of seed banks on the rate and direction of allele frequency changes as well as the maximum attainable frequency. In addition to testing previously proposed predictions on the effect of seed banks on genetic diversity, new patterns were found. Specifically, we determined that endospore formation may have the capacity to alter the direction of molecular evolution within a population. Stated inversely, the absence of endospore formation contributed to a substantial signal of divergent evolution for populations in energy-limited environments. This signal of divergence, alongside the observation that the absence of endospore formation provided a substantial fitness benefit, suggests that adaptation to energy-limited environments may be possible in the absence of a highly conserved life-history strategy. Though any such adaptation would likely be transitory, as the absence of endospore formation resulted in an increasingly strong fitness disadvantage as the degree of energy-limitation increased.

While our study offers evidence that the ability to form a seed bank alters the rate and direction of molecular evolution in *B. subtilis*, *spo0A* remains a confounding variable. The gene is a master regulator that can have pleiotropic effects on biofilm formation (Hamon and Lazazzera 2001; Pedrido *et al.* 2013; Dubnau *et al.* 2016), motility (Verhamme *et al.* 2009; Takada *et al.* 2014), and genetic competence (Hahn *et al.* 1995; Schultz *et al.* 2009; Mirouze *et al.* 2012). While we did not directly examine biofilm formation or motility, it is unlikely that they contributed toward adaptation as all replicate populations were maintained in well-aerated flasks in a constant shaker. However, the strength of correlations between mutation frequency trajectories remained higher in Δ*spo0A* across transfer regimes, a statistical pattern which suggests a lower rate of recombination (Supplementary Fig. 6). Such a reduction could occur if *spo0A* played a major role regulating genetic competence in our environmental conditions. Though the rate of recombination did not alter the distance between simulated distributions of mutation trajectory statistics for dormancy-capable and incapable populations (Supplementary Fig. 7). Finally, the main conclusions of our analyses of parallelism and divergent/convergent evolution were not affected by the inclusion of the *spo0A* regulon (Supplementary Figs. 9 and 14), suggesting that the pleiotropic effects of the gene were not so considerable that they were able to alter the distribution of mutation counts across genes or the degree of purifying selection.

This study tested lost-standing predictions and generated new questions for future research. Given that *Bacillus* incurs an environment-dependent fitness effect when endospore formation is removed, it is worth investigating the quantitative effects of mutation on endospore formation and, ultimately their effect on fitness. Given that limited evolutionary timescales can be obtained in experiments that mimic the environments where the ability to enter a dormant state has the greatest fitness benefit, alternative approaches may be necessary to probe the effect of mutation on endospore formation. One promising option is mass barcoding (e.g. RB-TnSeq), sidestepping the slow input of mutations via slow generation times by generating large mutant libraries, the frequencies of which can then be tracked (Wetmore *et al.* 2015). Such approaches have already been applied to spores in members of the genus *Streptomyces* (Wright and Vetsigian 2019) and could be leveraged to identify environment-dependent fitness effects as well as the mode in which traits, such as endospore formation, mediate fitness effects in *Bacillus* (Kinsler *et al.* 2020). Given that endospore formation is a complex trait with many loci that are likely interacting, it may be a suitable candidate to apply recently developed models that predict the form of the distribution of fitness effects when interactions between loci are prevalent (Reddy and Desai 2021).

## Data availability

Raw sequence data of Δ*spo0A* lines are available on the NCBI Sequencing Read Archive under BioProject ID PRJNA639642. Raw sequence data for WT lines were previously published (Shoemaker, Polezhaeva *et al.* 2021) and are available under BioProject ID PRJNA639414. Reproducible code to perform all simulations and analyses is available on GitHub under the repository: Bacillus_Evol_Timeseries. Processed data and annotations are available on Zenodo under the DOI: 10.5281/zenodo.5549311.

Supplemental material is available at *GENETICS* online.

## Supplementary Material

iyac071_Supplementary_Data
